# GSP-3D: generalizable 3D Gaussian Splatting with diffusion policy and world model for long-horizon bimanual manipulation

**DOI:** 10.3389/fnbot.2026.1867375

**Published:** 2026-08-31

**Authors:** Xukun Liu, Junhua Huang, Shenggang Wei, Liguo Hou, Junmou Qiao, Aifeng Liu, Hongsheng Huang, Fengjuan Xie

**Affiliations:** 1 Northwest Institute of Mechanical and Electrical Engineering, Xianyang, Shaanxi, China; 2 Jiangxi Guoke Defence Group Co., Ltd., Nanchang, Jiangxi, China

**Keywords:** 3D Gaussian Splatting, bimanual manipulation, diffusion policy, visuomotor learning control, world model

## Abstract

**Introduction:**

Learning robust visuomotor policies for bimanual manipulation remains challenging due to the stringent requirements for precise coordination between arms and the ability to generalize across diverse environmental conditions. Existing diffusion-based policies often suffer from temporally inconsistent action generation, while their reliance on sparse point cloud representations limits structural completeness and fails to capture future scene dynamics, hindering performance in long-horizon tasks.

**Methods:**

To address these limitations, we introduce GSP-3D, a unified framework that integrates generalizable 3D Gaussian Splatting (3DGS) with diffusion-based policy learning. GSP-3D comprises three key components: (1) a Generalizable Gaussian Regressor (GGR) that predicts 3D Gaussian primitives from a single RGB-D frame in real time; (2) a 3DGS-conditioned diffusion policy that aggregates Gaussians into compact latent representations, replacing point clouds with explicit geometric primitives; and (3) a transformer-based world model that forecasts future Gaussian sets and leverages prediction error as an auxiliary loss to enforce temporal consistency across actions.

**Results:**

We evaluate GSP-3D on the RoboTwin 2.0 benchmark across a range of bimanual manipulation tasks under both clean and domain-randomized conditions, including variations in lighting, backgrounds, and tabletop distractors. Experimental results show that GSP-3D consistently outperforms existing baseline methods, achieving higher success rates while maintaining minimal computational overhead.

**Discussion:**

These findings demonstrate that integrating explicit 3D Gaussian representations with diffusion policies offers an efficient and robust solution for long-horizon, temporally coherent bimanual manipulation, effectively addressing the generalization and consistency challenges that limit current approaches.

## 1 Introduction

Learning robust visuomotor policies for bimanual manipulation remains a fundamental challenge in neurorobotics. Unlike single-arm manipulation, bimanual tasks require precise temporal coordination between two manipulators while maintaining awareness of complex object interactions and long-horizon task dependencies. Achieving reliable performance becomes even more difficult in real-world environments where visual observations are subject to variations in lighting, background clutter, and object appearance. Therefore, effective policy learning demands not only accurate perception of 3D scene geometry but also the ability to anticipate future scene evolution under robot actions.

Recent diffusion-based visuomotor policies have demonstrated strong capability in modeling multimodal action distributions and handling diverse manipulation behaviors. However, existing approaches commonly rely on sparse geometric representations such as point clouds, which often fail to capture complete scene structure and provide limited information about object surfaces and spatial relationships. Moreover, diffusion policies are typically optimized solely through behavior imitation objectives, lacking explicit constraints on future scene dynamics. As a result, generated actions may exhibit temporal inconsistency and physically implausible transitions, particularly in challenging bimanual manipulation scenarios where coordinated interactions between both arms and manipulated objects are essential.

Meanwhile, advances in 3D scene representation, especially 3D Gaussian Splatting, have enabled efficient reconstruction and rendering of explicit scene geometry. Despite its success in computer vision and graphics, the potential of 3DGS for robotic policy learning remains largely unexplored. Furthermore, although world models have shown promise in capturing predictive dynamics and improving decision-making, their integration with diffusion-based imitation learning has received limited attention. Consequently, an important research gap remains: how to jointly leverage explicit 3D scene representations and predictive world modeling to improve the robustness, temporal consistency, and generalization of diffusion-based visuomotor policies for bimanual manipulation.

To address this challenge, we propose GSP-3D, a novel framework that unifies generalizable 3D Gaussian scene representation, diffusion policy learning, and predictive world modeling within a single architecture. Specifically, GSP-3D consists of three key components. First, a Generalizable Gaussian Regressor (GGR) predicts 3D Gaussian primitives directly from a single RGB-D observation, eliminating costly scene-specific optimization and enabling real-time deployment. Second, a 3DGS-conditioned diffusion policy aggregates Gaussian primitives through sparse voxelization and cross-attention mechanisms to obtain compact yet expressive scene representations, replacing sparse point-cloud inputs with structurally complete geometric information. Third, a transformer-based 3DGS world model predicts future Gaussian states conditioned on robot actions, and its prediction error is introduced as an auxiliary regularization objective during policy learning. This mechanism encourages temporally coherent action generation and physically plausible scene transitions.

The main scientific contributions of this work are three fold: (1) we introduce a unified framework that integrates generalizable 3D Gaussian Splatting with diffusion-based visuomotor policy learning for bimanual manipulation; (2) we propose a 3DGS-based predictive world model that regularizes policy learning through future scene forecasting, which helps improve temporal consistency and physical realism; and (3) we show that explicit Gaussian scene representations can enhance policy robustness and generalization under diverse visual perturbations.

We evaluate GSP-3D on the RoboTwin 2.0 benchmark across multiple challenging bimanual manipulation tasks under both standard and domain-randomized settings, including variations in lighting conditions, background textures, and tabletop distractors. Experimental results demonstrate that GSP-3D compares favorably against baseline methods, achieving higher task success rates, lower performance variance, and improved robustness while introducing only minimal computational overhead.

## 2 Related work

**Diffusion-based visuomotor policies:** Diffusion models have recently emerged as a powerful paradigm for robot policy learning, framing action generation as a conditional denoising process. Early work, such as Diffusion Policy (Chi et al., 2025), demonstrated that diffusion models can effectively capture multimodal action distributions and improve robustness over standard behavior cloning. Subsequent extensions have introduced temporal action chunking and receding-horizon control (Janner et al., 2022; Ajay et al., 2022), enabling long-horizon manipulation tasks. DP3 further enhances scalability by incorporating point cloud-based conditioning for 3D perception (Ze et al., 2024), while other approaches integrate transformer-based denoisers to improve temporal reasoning (Chen et al., 2021; Reed et al., 2022).

Recent studies have also explored improvements to diffusion policies under real-world uncertainties, including robustness to visual perturbations (Hansen et al., 2021), domain randomization (Tobin et al., 2017), and efficient sampling strategies (Song et al., 2020). Despite these advances, most existing methods rely on sparse geometric representations, such as point clouds or implicit visual features, which often lack structural completeness and temporal consistency. In contrast, GSP-3D uses explicit 3D Gaussian primitives for richer geometric conditioning and introduces a world model auxiliary loss to enforce temporal consistency.

**3D scene representation for robotics:** Accurate 3D scene understanding is critical for robotic manipulation. Traditional approaches have relied on point clouds (Charles et al., 2017; Qi et al., 2017), voxel grids (Shamil and Alwin, 2025), or implicit neural representations such as NeRF (Gao et al., 2022; Lin and Xu, 2024). While NeRF-based methods provide high-fidelity rendering, their high computational cost limits their applicability to real-time robotic systems (Ye et al., 2025).

Recently, 3D Gaussian Splatting has emerged as an efficient alternative for real-time rendering and explicit geometry representation (Kerbl et al., 2023). Extensions of 3DGS have explored dynamic scene reconstruction (Luiten et al., 2024), large-scale environments (Shu et al., 2023), and generalizable radiance field learning (Yen-Chen et al., 2021). Several works have attempted to bridge 3DGS with robotics for perception and mapping (Sucar et al., 2021; Zhu et al., 2022), demonstrating improved efficiency over NeRF-based pipelines. However, conventional 3DGS requires per-scene optimization, making it impractical for online policy learning. GSP-3D introduces a Generalizable Gaussian Regressor (GGR) that predicts 3D Gaussians from a single RGB-D frame in real time.

**World models and predictive dynamics learning:** Learning predictive world models has long been a central topic in robotics and reinforcement learning. Classical approaches model system dynamics explicitly using state transition functions (Deisenroth and Carl, 2011; Nagabandi et al., 2018), while recent works leverage deep neural networks for latent dynamics prediction (Matsuo et al., 2022; Hafner et al., 2019).

In visuomotor policy learning, world models have been used to improve sample efficiency and long-term reasoning (Hafner et al., 2020a,b). Latent imagination-based methods (Guei et al., 2026) and transformer-based dynamics models have further enhanced temporal modeling capabilities. More recent approaches incorporate predictive consistency losses into policy learning, improving stability and robustness (Shafiullah et al., 2022; Seo et al., 2021). However, despite these advances, most world models operate in low-dimensional latent spaces or point cloud representations, which often fail to capture fine-grained geometry and physical interactions. While prior world models operate in low-dimensional latent spaces, GSP-3D introduces a 3DGS-based world model that predicts future Gaussian states and uses prediction error as an auxiliary loss to regularize diffusion policy training.

Bimanual manipulation presents additional challenges compared to single-arm control due to the need for coordination, synchronization, and collision avoidance. Early approaches relied on analytical control and task decomposition (Sa et al., 2026), while learning-based methods have increasingly dominated recent research (Qi et al., 2025; Özkurt et al., 2026).

**Bimanual manipulation and coordination learning:** Imitation learning methods such as ACT (Zhao et al., 2023) and transformer-based policies (Brohan et al., 2022) have shown promising results in coordinated dual-arm tasks. Large-scale datasets and simulation platforms, such as RoboTwin (Chen et al., 2025), have further enabled scalable training and evaluation of bimanual policies. Recent works have also explored role assignment, temporal coordination, and shared latent representations for multi-arm systems (Huang et al., 2022; Motoda et al., 2025).

## 3 Method

**Figure 1:**
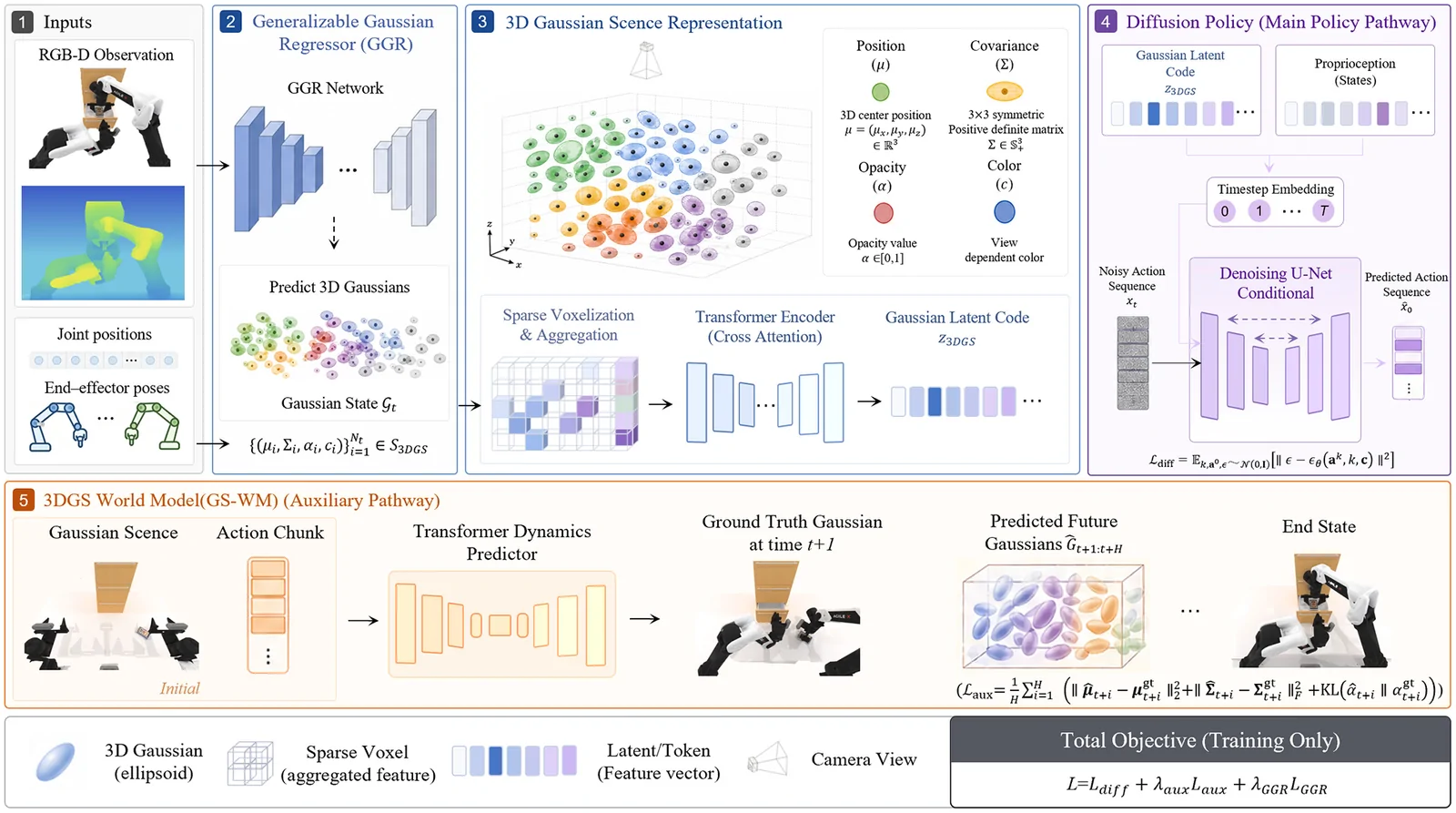
Overview of the proposed GSP-3D framework. The framework consists of a perception module (GGR), a main diffusion policy pathway, and an auxiliary world-model pathway for training regularization. Module 1: a single RGB-D frame is input to the system. Module 2: the generalizable Gaussian Regressor (GGR) predicts per-pixel 3D Gaussian primitives (position μ, covariance Σ, opacity α, color *c*) and is trained with reconstruction loss $L_{GGR}$. Module 3: predicted Gaussians are aggregated via sparse voxelization and cross-attention into a compact latent *z*_3*DGS*_. Module 4 (Main pathway): a denoising U-Net ϵ_θ_ takes noisy actions *a*^*k*^, 3DGS latent *z*_3*DGS*_, proprioception *q*, and task embedding *e*_*robot*_ to predict noise, optimized by diffusion loss $L_{diff}$. Modules 5 (Auxiliary pathway): a transformer-based world model (GS-WM) predicts future Gaussian states ${\hat{G}}_{t+1:t+H}$ from past Gaussians and action chunks, auxiliary loss $L_{aux}$ computes the discrepancy between predicted and ground-truth future Gaussians (positions, covariances, opacities) to enforce temporal consistency. Total training objective $L_{total}=L_{diff}+\lambda_{aux}L_{aux}+\lambda_{GGR}L_{GGR}$. The auxiliary pathway is used only during training; at test time, the policy executes actions from current observations alone.

We present GSP-3D, a novel framework that integrates generalizable 3D Gaussian Splatting into diffusion-based visuomotor policy learning for robust bimanual manipulation. Unlike prior works that rely on sparse point clouds or RGB-D images, GSP-3D introduces a lightweight Gaussian Regressor that dynamically reconstructs scene geometry as 3D Gaussian primitives in real time. Furthermore, we propose a 3DGS-based world model that predicts future multi-frame point cloud states, providing an auxiliary regularization loss that enforces temporal consistency and physical plausibility during diffusion training. The overall architecture, illustrated in Figure 1, integrates these components into an end-to-end trainable system.

### 3.1 Preliminaries

**Diffusion policy:** Diffusion Policy formulates action generation as a conditional denoising process. Given a history of observations and robot states, the policy learns to reverse a forward diffusion process that gradually corrupts action chunks with Gaussian noise.

Formally, let *a*^0^∈*R*^*H*×*a*^ denote a sequence of *H* future actions, where *H* is the action chunk length, and *a* is the action dimensionality. Let *k* indicate the diffusion timestep index, where *K* is the total number of diffusion steps. Let ϵ~(0, **I**) denote the standard Gaussian noise, where **I** is the identity matrix of appropriate dimension. ϵ_θ_ is the denoising network with learnable parameters θ, which predicts the noise added to **a**^*k*^. Let *c* denote conditioning information, which includes visual features and robot proprioception.

The forward process adds noise over *K* steps, and the reverse process learns a denoising network ϵ_θ_ to predict the noise. The training objective is Equation 1:


$$\begin{array}{l} \begin{array}{c} L_{diff}=E_{k,{\text{a}}^{0},\epsilon N\left(0,\text{I}\right)}\left[∥\epsilon -\epsilon_{\theta }\left({\text{a}}^{k},k,\text{c}\right){∥}^{2}\right] \end{array} \end{array}$$ (1)

In standard DP3, the visual conditioning is derived from sparse point clouds encoded via a lightweight MLP.

**3D Gaussian Splatting:** 3DGS represents a scene as a set of anisotropic 3D Gaussians, each parameterized by mean position μ∈ℝ^3^, mean position in world coordinates. Covariance matrix **Σ**∈ℝ^3 × 3^, covariance matrix. Opacity α∈[0, 1], opacity, controlling the primitive's transparency and spherical harmonic coefficients for view-dependent color. Rendering is performed via differentiable rasterization, enabling real-time novel view synthesis. Unlike implicit representations, 3DGS offers explicit geometry, fast inference, and compatibility with physics-based reasoning—properties highly desirable for robotic manipulation. However, conventional 3DGS requires per-scene optimization, which is impractical for online policy learning. Our key insight is to train a generalizable Gaussian Regressor that directly predicts Gaussian primitives from single-view observations, enabling real-time dynamic 3D reconstruction without test-time optimization.

**Generalizable Gaussian regressor (GGR):** We design a lightweight encoder-decoder that predicts per-pixel 3D Gaussian parameters from a single RGB-D frame, producing a dynamic 3D scene representation.

**Input and output:** Given a single RGB-D image $I\in {\mathbb{R}}^{H\times W\times 4}$ (a single RGB-D image consisting of RGB channels and a depth channel, where *H* and *W* are the height and width in pixels) GGR predicts a set of 3D Gaussians $G={\left\{\left(\mu_{i},\Sigma_{i},\alpha_{i},{\text{c}}_{i}\right)\right\}}_{i=1}^{N}$ that reconstructs the visible scene.

**Architecture:** GGR adopts a U-Net-style architecture with three stages. A shared encoder first processes the RGB-D input to extract multi-scale spatial features **F**∈ℝ^*H*′ × *W*′ × *C*^(where *C* is the number of feature channels). Subsequently, a depth-to-point cloud back-projection module leverages camera intrinsics to unproject each pixel into 3D space, generating an initial point cloud $P_{init}={\left\{\left(p_{j},f_{j}\right)\right\}}_{j=1}^{H^{\prime }W^{\prime }}$, where ${\text{p}}_{j}\in {\mathbb{R}}^{3}$ denotes the 3D position and **f**_*j*_ the associated feature vector rom **F**. A lightweight point-based decoder then regresses Gaussian parameters for each point (Equation 2):


$$\begin{array}{l} \begin{array}{c} \left(\mu_{i},\Sigma_{i},\alpha_{i},{\text{c}}_{i}\right)=MLP_{dec}\left({\text{f}}_{i}\right) \end{array} \end{array}$$ (2)

where the covariance **Σ**_*i*_ is parameterized as a scaled rotation $\Sigma_{i}={\text{R}}_{i}{\text{S}}_{i}{\text{S}}_{i}^{T}{\text{R}}_{i}^{T}$ with scale ${\text{s}}_{i}\in {\mathbb{R}}^{3}$ and quaternion ${\text{q}}_{i}\in {\mathbb{R}}^{4}$. The opacity α_*i*_ is passed through a sigmoid activation, and color coefficients are normalized to [−1, 1].

**Training with differentiable rendering:** GGR is trained end-to-end with a differentiable rasterizer that renders predicted Gaussians back to image space. The rendering process aggregates Gaussian contributions per pixel via alpha compositing (Equation 3):


$$\begin{array}{l} \begin{array}{c} J_{render}\left(\text{x}\right)=\overset{N}{\underset{i=1}{\sum }}\;{\text{c}}_{i}\alpha_{i}\exp\left(-\frac{1}{2}{\left(\text{x}-\mu_{i}^{2D}\right)}^{T}\Sigma_{i}^{2D^{-1}}\left(\text{x}-\mu_{i}^{2D}\right)\right) \end{array} \end{array}$$ (3)

$$\begin{array}{l} \begin{array}{c} \overset{i-1}{\underset{j=1}{\prod }}\;\left(1-\alpha_{j}\right) \end{array} \end{array}$$

where $\mu_{i}^{2D}\in {\mathbb{R}}^{2}$ and $\Sigma_{i}^{2D}\in {\mathbb{R}}^{2\;\times \;2}$ are the projected Gaussian parameters onto the image plane via the camera projection function π(•). The combined training loss is Equation 4:


$$\begin{array}{l} \begin{array}{c} L_{GGR}=\lambda_{1}∥J_{render}-I_{gt}{∥}_{1}+\lambda_{2}L_{SSIM}\left(J_{render},J_{gt}\right)+\lambda_{3}∥ \end{array} \end{array}$$ (4)

$$\begin{array}{l} \begin{array}{c} D_{render}-D_{gt}{∥}_{2}^{2} \end{array} \end{array}$$

where the first two terms supervise RGB reconstruction and the third term supervises depth rendering. The training data is generated from RoboTwin-OD under diverse domain-randomized conditions including cluttered scenes, varied lighting, and background textures.

**Robustness of GGR under domain randomization:** To ensure reliable Gaussian prediction under visual perturbations, GGR incorporates several complementary mechanisms. The U-Net encoder is trained with aggressive data augmentation including random color jitter, Gaussian blur, and simulated depth sensor noise, forcing the feature extractor to learn illumination-invariant representations. The depth-to-point cloud back-projection uses median-filtered depth maps to mitigate outlier depth readings caused by reflective surfaces or sensor artifacts. The differentiable rendering loss implicitly regularizes the predicted Gaussians by enforcing multi-view photometric and geometric consistency; even under challenging lighting or texture variations, the rendering error gradient encourages the regressor to produce Gaussians that reconstruct the scene faithfully. Regarding policy network tolerance to residual artifacts, the subsequent sparse voxelization and max-pooling aggregation naturally discard outlier Gaussians with low opacity or inconsistent covariance. The diffusion policy is trained on diverse domain-randomized data (random lighting, backgrounds, and distractors), which inherently teaches the denoising network to treat moderate Gaussian prediction errors as part of the observation noise distribution.

### 3.2 Diffusion policy with 3DGS conditioning

To incorporate 3DGS into the diffusion policy within GSP-3D, we aggregate the predicted Gaussians into a compact Gaussian latent code that serves as visual conditioning.

**Gaussian feature aggregation:** Directly feeding all Gaussians into the diffusion network is computationally prohibitive. We therefore introduce a sparse 3D convolution backbone that operates on voxelized Gaussian features. Specifically, we voxelize the 3D space at multiple resolutions {*r*_1_, *r*_2_, …, *r*_*L*_} (multiple voxel resolutions), *voxel*_*l, m*_ (the m-th voxel at resolution *r*_*l*_) and *MLP*_*agg*_ (a small MLP that maps a single Gaussian to a feature vector of dimension *D*_*v*_).

For each voxel at resolution *r*_*l*_, aggregate the Gaussian parameters inside via probabilistic max-pooling (Equation 5):


$$\begin{array}{l} \begin{array}{c} {\text{v}}_{l,m}=\underset{i\in voxel_{l,m}}{\max}\left\{{\text{MLP}}_{\text{agg}}\left(\mu_{i},\Sigma_{i},\alpha_{i},{\text{c}}_{i}\right)\right\} \end{array} \end{array}$$ (5)

producing a multi-scale feature volume **V** = {_**v**_*l, m*_}*l, m*_. This volume is then flattened and projected to a compact latent vector ${\text{z}}_{3DGS}\in {\mathbb{R}}^{D_{z}}$ via a transformer encoder with cross-attention (Equation 6):


$$z_{3\text{DGS}}=\text{CrossAttn}\left(Q,\text{Flatten}\left(V\right)\right)$$ (6)

where Flatten(**·**) denotes operation that concatenates all **v**_*l, m*_ into a sequence of length $\sum_{l}\left(\frac{H^{\prime }}{r_{l}}\cdot \frac{W^{\prime }}{r_{l}}\right)$, $\text{Q}\in {\mathbb{R}}^{D_{z}\times D_{v}}$ indicate learnable query vectors, with *D*_*z*_ being the latent dimension.

**Conditioning injection:** The diffusion denoising network ϵ_θ_ conditions on three sources: the 3DGS latent **z**_3*DGS*_, the robot proprioception $\text{q}\in {\mathbb{R}}^{d_{q}}$, and the diffusion timestep *k*. We inject ${\text{z}}_{3DGS}\in {\mathbb{R}}^{D_{z}}$ via cross-attention layers within the denoising UNet, while **q** is concatenated with the noisy action sequence. The complete conditioning is **c** = {**z**_3*DGS*_, **q**}. The conditional reverse process is thus Equation 7:


$$\begin{array}{l} \begin{array}{c} {\text{a}}^{k-1}=\frac{1}{\sqrt{1-\beta_{k}}}\left({\text{a}}^{k}-\frac{\beta_{k}}{\sqrt{1-{\overset{-}{\alpha }}_{k}}}\epsilon_{\theta }\left({\text{a}}^{k},k,{\text{z}}_{3DGS},\text{q}\right)\right)+\sigma_{k}\text{z} \end{array} \end{array}$$ (7)

where β_*k*_∈(0, 1) denotes forward process variance schedule, ${\overset{-}{\alpha }}_{k}=\prod_{s=1}^{k}\left(1-\beta_{s}\right)$ indicates cumulative product, σ_*k*_ reverse process noise scale (typically set to $\sqrt{\beta_{k}}$).

### 3.3 3DGS-based world model as auxiliary regularization

A key limitation of standard diffusion policies is that they learn action distributions without explicit modeling of future scene dynamics. This often leads to temporally inconsistent actions that produce physically implausible state transitions. To address this, we introduce a 3DGS-based World Model (GS-WM) that predicts future point cloud states given current observations and actions, and uses its prediction error as an auxiliary regularization loss within GSP-3D.

**State space definition:** Before presenting the world model architecture, we formally define the state space in which GS-WM operates. Unlike prior world models that operate in low-dimensional latent spaces or point cloud spaces, GS-WM operates in the 3D Gaussian primitive state space, which we denote as $S_{3\text{D}G\text{S}}$. This state space is defined as the set of all possible collections of 3D Gaussian primitives that represent scene geometry (Equation 8):


$$\begin{array}{l} \begin{array}{c} S_{3DGS}=\left[\begin{array}{c} G={\left\{\left(\mu_{i},\Sigma_{i},\alpha_{i},{\text{c}}_{i}\right)\right\}}_{i=1}^{N}| \end{array}\right] \end{array} \end{array}$$ (8)

$$\begin{array}{l} \begin{array}{c} N\in \mathbb{N},\mu_{i}\in {\mathbb{R}}^{3},\Sigma_{i}\in S_{++}^{3},\alpha_{i}\in \left[0,\;1\right],{\text{c}}_{i}\in {\mathbb{R}}^{3} \end{array} \end{array}$$

where $S_{++}^{3}$ denotes the set of 3 × 3 symmetric positive definite matrices, and **c**_*i*_ represents the color coefficients (RGB). The number of Gaussians *N* is not fixed and varies with scene complexity.

**World model architecture:** GS-WM is a conditional dynamics model that learns a transition function $T:S_{3DGS}\text{×}A\to S_{3DGS}$, where A denotes the action space (target joint positions for both arms). Given a history of past Gaussian states and robot actions, GS-WM predicts future Gaussian states.

Let $G_{t}={\left\{\left(\mu_{t}^{i},\Sigma_{t}^{i},\alpha_{t}^{i},{\text{c}}_{t}^{i}\right)\right\}}_{i=1}^{N}$ denote the set of Gaussians at time *t*. Given a history of *T* past Gaussian sets and an action chunk **a**_*t*:*t*+*H*−1_, GS-WM predicts the future Gaussian sets via a transformer-based dynamics predictor (Equation 9):


$$\begin{array}{l} \begin{array}{c} {\hat{G}}_{t+1:t+H}={\text{Transformer}}_{WM}\left(G_{t-T:t},{\text{a}}_{t:t+H-1}\right) \end{array} \end{array}$$ (9)

To enable tokenization of Gaussian sets, we first project each Gaussian into a token embedding (Equation 10):


$$\begin{array}{l} \begin{array}{c} {\text{g}}_{t}^{i}={\text{MLP}}_{\text{token}}\left(\mu_{t}^{i},\;vec\left(\Sigma_{t}^{i}\right),\alpha_{t}^{i},{\text{c}}_{t}^{i}\right)\in {\mathbb{R}}^{D_{g}} \end{array} \end{array}$$ (10)

Where *vec* (·) denotes vectorization operator that flattens the 3 × 33 covariance matrix into a 9-dimensional vector, *D*_*g*_ indicates token embedding dimension.

This yields a token sequence ${\left\{{\text{g}}_{t}^{i}\right\}}_{i=1}^{N}$ at each timestep. The transformer models temporal dependencies via causal attention (Equation 11):


$$h_{t+1}^{i}=\text{Attn}\left(g_{t}^{i},{\left\{g_{t}^{j}\right\}}_{j=1}^{N},a_{t}\right)$$ (11)

and outputs residual updates to the Gaussian parameters (Equation 12):


$$\begin{array}{l} {\hat{\mu }}_{t+1}=\mu_{t}^{i}+\Delta \mu_{t}^{i}, \end{array}$$ (12)

$$\begin{array}{l} {\hat{\Sigma }}_{t+1}^{i}=\Sigma_{t}^{i}+\Delta \Sigma_{t}^{i}, \end{array}$$

$$\begin{array}{l} {\hat{\alpha }}_{t+1}^{i}=\sigma \left(\sigma^{1}\left(\alpha_{t}^{i}\right)+\Delta \alpha_{t}^{i}\right), \end{array}$$

$$\begin{array}{l} {\hat{\text{c}}}_{t+1}^{i}={\text{c}}_{t}^{i}+\Delta {\text{c}}_{t}^{i} \end{array}$$

where σ(·) = 1/(1+*e*^−·^) is the sigmoid function (applied to keep opacities in [0, 1]), and σ^−1^ is its inverse (logit function). The residual updates $\Delta \mu_{t}^{i},\;\Delta \Sigma_{t}^{i},\;\Delta \alpha_{t}^{i},\;\Delta {\text{c}}_{t}^{i}$ are predicted by the transformer decoder.

**Training the world model:** GS-WM is trained separately from the diffusion policy using a supervised prediction objective on offline data collected from RoboTwin 2.0. The training data consists of tuples $\left(G_{t-T:t},{\text{a}}_{t:t+H-1},G_{t+1:t+H}\right)$ extracted from expert demonstrations. The training loss is Equation 13:


$${ℒ}_{WM}=\frac{1}{H}\sum_{i=1}^{H}(∥{\hat{\mu }}_{t+i}-\mu_{t+i}{∥}_{2}^{2}+∥{\hat{\Sigma }}_{t+i}-\Sigma_{t+i}{∥}_{F}^{2}$$

$$\begin{array}{l} +BCE({\hat{\alpha }}_{t+i},\alpha_{t+i})+∥{\hat{c}}_{t+i}-c_{t+i}{∥}_{2}^{2}) \end{array}$$ (13)

where BCE denotes binary cross-entropy (since opacities are bounded in [0, 1]) and ∥·∥_*F*_ is the Frobenius norm for covariance matrices. The world model is trained until convergence and then frozen during policy learning.

**Auxiliary regularization loss:** During policy training, we freeze GS-WM and compute the prediction error between the predicted future Gaussians and the ground truth Gaussians extracted from subsequent observations. Critically, this auxiliary loss operates in the same state space $S_{3DGS}$ as the world model (Equation 14):


$$\begin{array}{l} {ℒ}_{aux}=\frac{1}{H}\overset{i=1}{\underset{H}{\sum^{\text{​}}}}(∥{\hat{\mu }}_{t+i}-\mu_{t+i}^{gt}{∥}_{2}^{2}+∥{\hat{\Sigma }}_{t+i}-\Sigma_{t+i}^{gt}{∥}_{F}^{2}\\ \;+\;\;\;\;\;\;KL({\hat{\alpha }}_{t+i}∥\alpha_{t+i}^{gt})) \end{array}$$ (14)

Where $\mu_{t+i}^{gt},\Sigma_{t+i}^{gt},\alpha_{t+i}^{gt}$ denotes ground-truth Gaussian parameters obtained from the RGB-D observations at time *t*+*i* via the same GGR (applied offline to all training frames).

**Training-only auxiliary loss and test-time deployment:** It is important to clarify that the auxiliary loss $L_{aux}$ is used exclusively during training, not during real-world deployment. The ground truth Gaussians $\alpha_{t+i}^{gt}$ are obtained offline by applying the pre-trained GGR to each frame of the expert demonstration dataset, which is feasible because complete RGB-D trajectories are available in the offline training phase. At test time, the diffusion policy executes actions based solely on current observations (via GGR) and proprioception, without requiring any future ground truth or online prediction from the world model. The role of${\;L}_{aux}\;$is purely to regularize the policy's training objective, encouraging the policy to favor actions that lead to temporally coherent scene transitions as modeled by GS-WM. The auxiliary loss simply provides an additional training signal, the policy learns to internalize temporal consistency directly into its action generation process, rather than relying on the world model during execution.

The KL divergence term for opacities is defined as Equation 15:


$$\begin{array}{l} \begin{array}{c} \text{KL}\left(\hat{\alpha }∥\alpha^{gt}\right)=\hat{\alpha }\log\frac{\hat{\alpha }}{\alpha^{gt}}+\left(1-\hat{\alpha }\right)\log\frac{1-\hat{\alpha }}{1-\alpha^{gt}} \end{array} \end{array}$$ (15)

**Intuition and effect:** By operating directly in $S_{3DGS}$, GS-WM can capture fine-grained geometric and temporal scene dynamics that would be lost in lower-dimensional latent spaces. For bimanual manipulation, this is particularly important because: (1) the spatial relationship between the two arms and the manipulated objects is explicitly represented via Gaussian positions μ_*i*_; (2) object deformations or pose changes are captured via changes in covariance Σ_*i*_; and (3) occlusions and object disappearances are naturally handled via opacity α_*i*_ approaching zero. Minimizing $L_{aux}$ encourages the diffusion policy to generate actions that lead to physically coherent future scenes according to the learned world model, improving temporal consistency and reducing physically implausible action sequences.

It is worth clarifying the intended role of GS-WM within the proposed framework. 3DGS is fundamentally a radiance field representation rather than a physical dynamics model. GS-WM is not designed to replace a rigorous physics simulator or to guarantee physically accurate predictions under complex object interactions such as deformation or collision. Instead, its purpose is to provide an additional regularization signal that encourages the diffusion policy to favor actions leading to temporally coherent scene transitions as captured by the training data. The GS-WM learns empirical transition patterns from expert demonstrations, which implicitly encode physical constraints (e.g., objects move continuously, occlusions evolve smoothly). The auxiliary loss penalizes large or abrupt changes in Gaussian parameters that deviate from these learned patterns, thereby reducing the likelihood of physically implausible action sequences. For scenarios involving complex interactions such as handing over or stacking, the expert demonstrations naturally contain examples of successful coordination; the GS-WM captures the statistical regularities of these transitions without explicitly modeling collision or dynamics. Incorporating a more principled physical inductive bias, such as a collision detection module or a rigid-body dynamics prior, could further improve physical realism, which we leave as future work.

### 3.4 Integration with embodiment-aware adaptation

To enable GSP-3D to handle a variety of bimanual manipulation tasks within a fixed dual-arm system, we incorporate task-conditioned adaptation mechanisms following the design principles of RoboTwin 2.0. Each object in the RoboTwin-OD dataset is annotated with multiple candidate grasp poses covering diverse approach directions (e.g., top-down, side, diagonal) and grasp axes. These affordances are encoded as additional Gaussian attributes during GGR prediction, enriching the 3DGS representation with task-relevant interaction priors (Equation 16):


$$\begin{array}{l} \begin{array}{c} {\tilde{\text{c}}}_{i}={\text{c}}_{i}⊕{\text{MLP}}_{\text{aff}}\left({\text{a}}_{\text{grasp}}^{i}\right) \end{array} \end{array}$$ (16)

where ${\text{a}}_{grasp}^{i}\in {\mathbb{R}}^{d_{grasp}}$ encodes the grasp affordance vector and ⊕ denotes concatenation.

During training, we condition the diffusion policy on a learnable task embedding ${\text{e}}_{robot}\in {\mathbb{R}}^{16}$ that captures the specific manipulation objective (e.g., lifting, handing over, stacking, insertion). The 3DGS representation remains task-agnostic, providing a shared geometric understanding across different scenarios, while the task embedding allows the policy to adapt its action distribution to the required coordination pattern (Equation 17):


$$\begin{array}{l} \begin{array}{c} \epsilon_{\theta }\left({\text{a}}^{k},k,{\text{z}}_{\text{3DGS}},\text{q},{\text{e}}_{\text{robot}}\right) \end{array} \end{array}$$ (17)

The complete training objective for GSP-3D integrates all components (Equation 18):


$$\begin{array}{l} \begin{array}{ccc} {\text{L}}_{\text{total}} & = & {\text{E}}_{k,{\text{a}}^{0},\epsilon }\left[∥\epsilon -\epsilon_{\theta }\left({\text{a}}^{k},k,{\text{z}}_{\text{3DGS}},\text{q},{\text{e}}_{\text{robot}}\right){∥}^{2}\right]\\ + & \lambda_{\text{aux}}\frac{1}{H}\overset{H}{\underset{i=1}{\sum }}\;D\left({\hat{G}}_{t+i},G_{t+i}^{gt}\right)+\lambda_{\text{GGR}}L_{\text{GGR}} \end{array} \end{array}$$ (18)

Where D(·,·) denotes the same distance function as defined in LauxLaux, λ_*GGR*_ is the hyperparameter weighting the GGR reconstruction loss.

This task-conditioned design enables robust adaptation across a range of bimanual tasks on a fixed dual-arm platform. By conditioning on the task embedding, the policy learns to adjust its coordination strategy—such as which arm initiates the grasp, how to sequence bimanual actions, and how to avoid inter-arm interference—without requiring task-specific engineering. The GS-WM further anticipates task-relevant dynamics: for tasks requiring fine synchronization (e.g., handing over an object), the model learns to predict when a planned action may lead to instability, allowing the diffusion policy to adjust accordingly.

## 4 Experiments

### 4.1 Experimental setup

**Figure 2:**
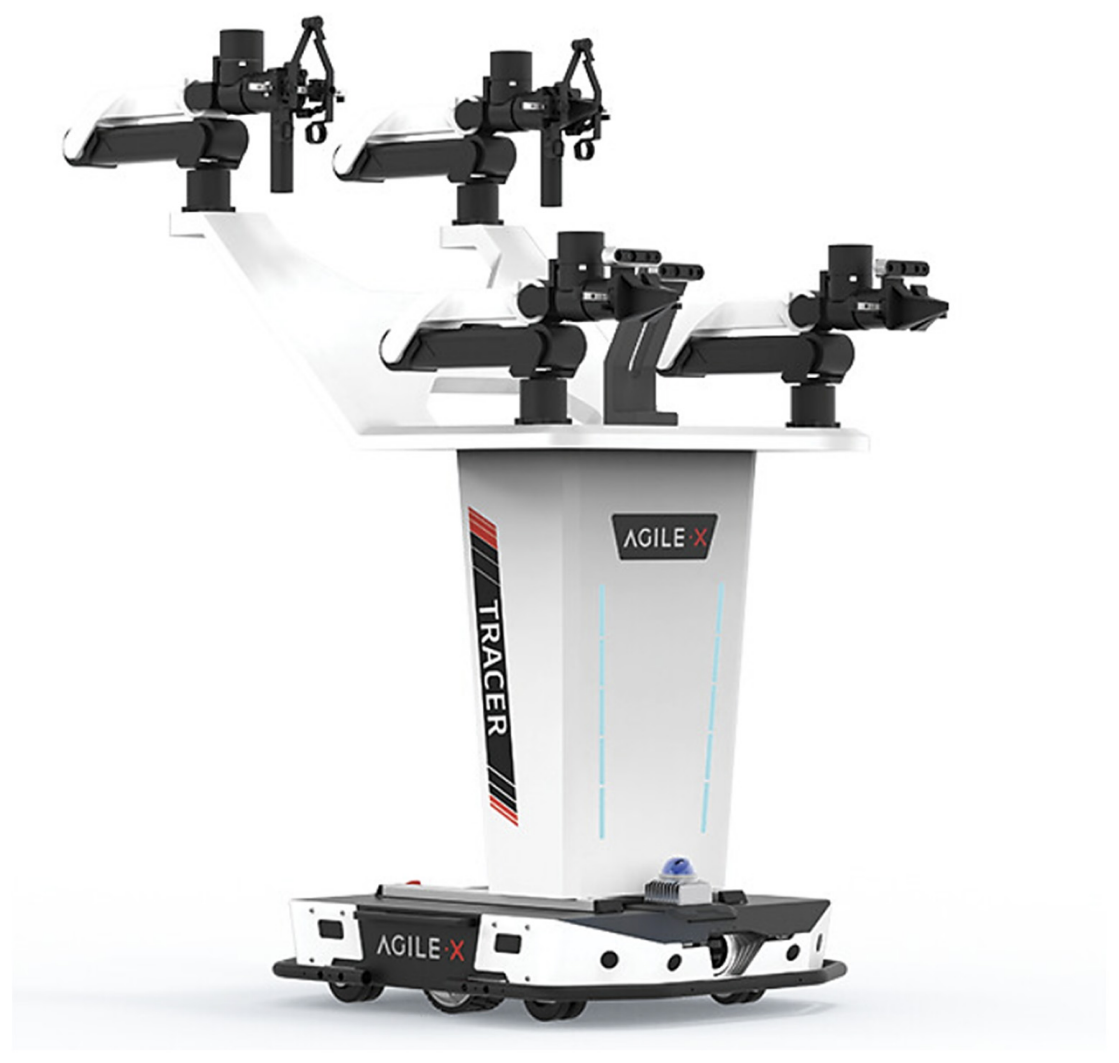
Dual-arm robotic platforms.

**Experimental platform:** We evaluate GSP-3D on the RoboTwin 2.0 platform, which provides a unified simulation environment and a scalable benchmark for bimanual robotic manipulation. Dual-arm evaluations are conducted using Aloha-AgileX platforms, as illustrated in the Figure 2.

**Data generation:** The training data used in this study were automatically generated using the built-in data generation pipeline of RoboTwin 2.0. This pipeline is a standard feature of the platform and is used as-is without modification. All methods evaluated in this paper—our GSP-3D and all baselines (DP3, DP, ACT, Pi0, and RDT)—were trained and tested on exactly the same set of expert demonstrations to ensure a fair and unbiased comparison. Our research focuses on the policy learning framework itself; therefore, we did not modify or customize the data generation process. Further details of the RoboTwin 2.0 data generation pipeline can be found in the original platform documentation.

Each expert demonstration was recorded in the RoboTwin 2.0 data collection framework and stored as a complete trajectory in HDF5 format. The simulator operates at 250 Hz (0.004 s per control step), while observations and actions are saved every 15 simulation steps, resulting in an effective sampling frequency of approximately 16.7 Hz. Depending on task complexity, each trajectory typically contains 100–250 recorded timesteps, corresponding to approximately 7–15 s of interaction. Observations consist of RGB images (640 × 480), depth images (640 × 480), robot proprioceptive states including joint positions (qpos, 14-dimensional) and end-effector poses, and the associated language instruction. Actions are represented as 14-dimensional joint position targets for the two 7-DoF arms (seven dimensions per arm), consistent with the standard Aloha-AgileX joint-space control interface.

For pre-processing, we follow a unified pipeline applied to all methods to ensure fair comparison. Each raw trajectory is temporally segmented into overlapping clips of length *T*_*obs*_+*T*_*pred*_, where *T*_*obs*_ = 3 (observation steps) and *T*_*pred*_ = 6 (action prediction steps). The segmentation stride is 1 timestep, yielding approximately 100–245 training samples per trajectory. We then apply per-dimension mean-std normalization to the action vectors: for each action dimension *d*, we compute the mean μ_*d*_ and standard deviation σ_*d*_ across all training samples and normalize actions as $a_{d}^{norm}=\left(a_{d}-\mu_{d}\right)/\left(\sigma_{d}+\epsilon \right)$. The same normalization statistics are applied to all baselines. For GSP-3D and DP3, point cloud or Gaussian inputs are derived from RGB-D images using camera intrinsics; no additional normalization is applied to geometric coordinates beyond centering on the robot workspace. All methods consume the same pre-processed observation-action pairs during training, and at test time, actions are denormalized back to raw joint position commands before execution.

**Baseline selection:** We select RDT, Pi0, ACT, DP, and DP3 as baselines because they represent the current state-of-the-art in visuomotor policy learning and have been previously evaluated on bimanual manipulation tasks within the RoboTwin 2.0 benchmark. Although these baselines employ different input modalities (e.g., RGB for DP, point clouds for DP3, language instructions for Pi0) and Pi0 uses a pre-trained vision-language backbone, all methods are trained and evaluated under strictly identical conditions in our study: the same 50 expert demonstrations per task, the same 14-dimensional joint position action space, the same domain-randomized evaluation settings (100 trials per task), and the same success criteria to ensure a fair comparison.

**On the fairness of cross-modality comparison:** We acknowledge that the baselines differ in their input modalities and that Pi0 benefits from large-scale pretraining, which may confer advantages in generalization. Our goal is not to claim a strictly controlled ablation of input types, but rather to evaluate how different *scene representations* (2D images, sparse point clouds, and our explicit 3D Gaussians) affect bimanual manipulation performance when integrated with modern policy architectures. P0′s pretrained backbone provides it with additional real-world prior knowledge, yet our GSP-3D, trained from scratch solely on RoboTwin 2.0 demonstrations, achieves higher success rates on most tasks. We interpret the comparison as evidence for the effectiveness of our proposed representation and architecture, rather than a modality-controlled ablation.

**Figure 3:**
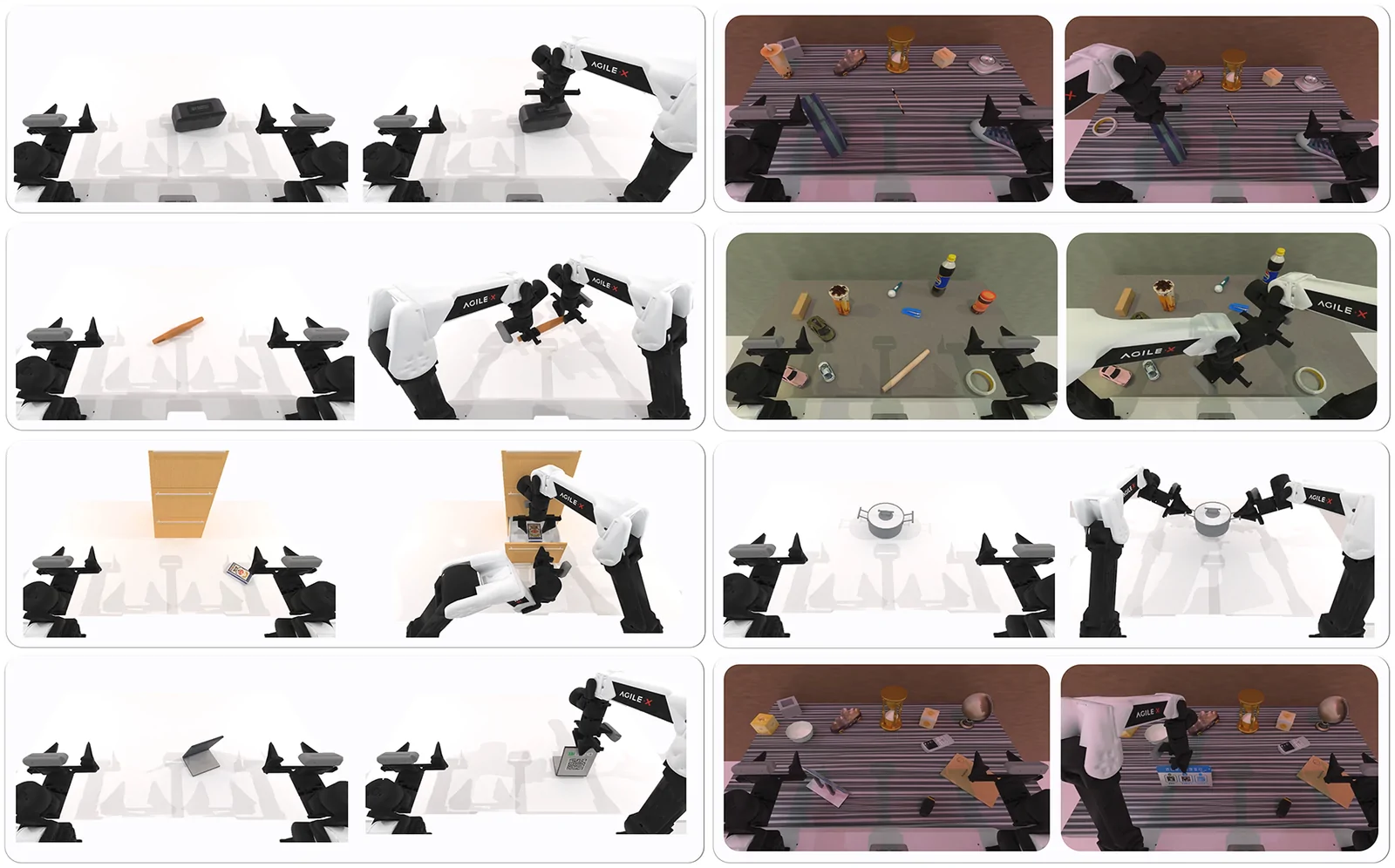
Illustrative collaborative bimanual tasks under clean and domain randomization.

**Task description:** In our experiments, the robot is required to complete a diverse set of bimanual manipulation tasks under varying initial conditions while avoiding collisions. The key challenges include spatial uncertainty of target objects, as well as environmental disturbances such as changes in lighting, background textures, and viewing distances during task execution (Figure 3).

### 4.2 Implementation details

To facilitate the replication of our results, this section describes the experimental environment, evaluation methodology, and key model parameters employed throughout this study.

**Evaluation settings:** All compared approaches are tested under identical simulation conditions to ensure a fair and unbiased comparison. For every task, we perform 100 independent trial runs and compute the average success percentage over the entire set. The experiments are run on a laptop featuring an NVIDIA GeForce RTX 4060 GPU (ROG Strix G8 Plus).

**Model hyperparameters:** The critical hyperparameters adopted for our primary model are fully specified below. A detailed summary of the configuration tailored to our dual-arm manipulation tasks is provided in Table 1.

**Table 1:** Hyperparameter configuration for GSP-3D.

Category	Hyperparameter	Value	Category	Hyperparameter	Value
Model architecture	Observation steps	3	Training	Learning rate	1e-4
	Action steps	6		Batch size	256
	Diffusion step embed dim	64		Optimizer	AdamW
	Encoder output dim	128		Weight decay	1e-6
	Down dims	[512, 1,024, 2,048]		LR scheduler	Cosine
	Kernel size	5		Seed	42
Platform configuration	Simulator version	release version 2025 Oct 27	Task configuration	random_background	true
	Simulation timestep	6,000		cluttered_table	true
	Robot model	Aloha-AgileX		random_table_height	0.03
	Camera type	D435		random_light	true
	Image resolution	640 × 480		crazy_random_light_rate	0.02
	Depth pre-processing	Median filtering		language_num	100

**Data flow across training stages:** To further clarify the data flow across different training stages, we specify the exact observation and action structure used for each component of GSP-3D. For the Generalizable Gaussian Regressor (GGR) training, each training sample consists of a single RGB-D frame (640 × 480) as input and the corresponding rendered RGB-D target from the same viewpoint. For diffusion policy training, each sample contains a history of *T*_*obs*_ = 3 consecutive RGB-D frames (passed through GGR to obtain 3D Gaussians) and a history of *T*_*obs*_ = 3 proprioceptive states (joint positions), with the target being the next *T*_*pred*_ = 6 action steps. For world model training, each sample includes a sequence of *T*_*obs*_ = 3 Gaussian sets $G_{t-2:t}$ and action chunk **a**_*t*:*t*+5_ as input, and the next *T*_*pred*_ = 6 Gaussian sets $G_{t+1:t+6}$ as prediction targets. All three training stages use the same underlying trajectory data but construct input-output pairs differently according to their respective objectives. Raw trajectories are never used directly by any policy; only pre-processed and normalized samples are fed into training.

### 4.3 Experimental results

**Figure 4:**
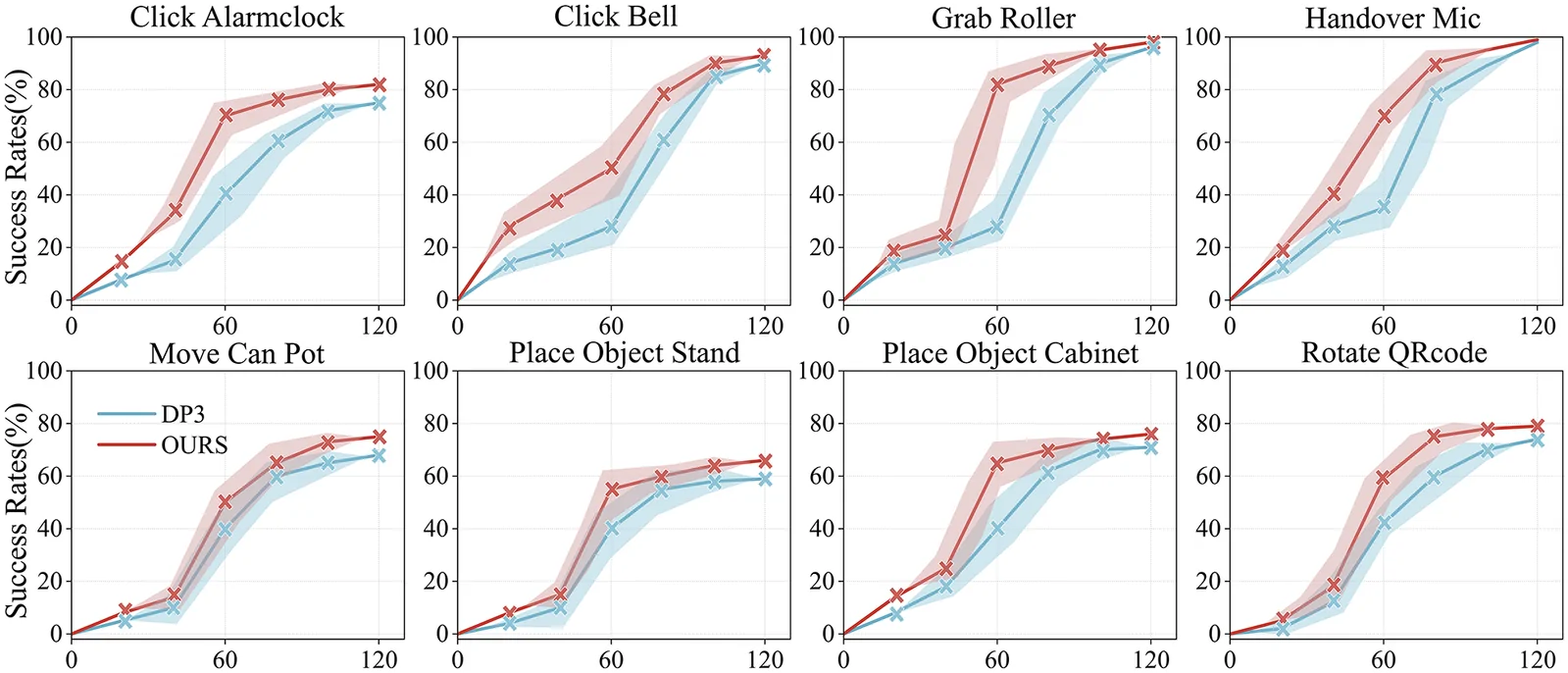
Success rate vs. training epochs for GSP-3D and DP3 under the demo clean setting. Each curve is averaged over three random seeds. Results are shown for all eight tasks.

**Learning efficiency:** We tracked the task success rate throughout the training process to evaluate the learning progress and convergence behavior of our proposed method across different experiments. The evolution of the success rate over training epochs is summarized for all evaluated tasks, demonstrating the superior learning efficiency and convergence of our approach in coordinated bimanual control tasks (Figure 4).

**Computational complexity analysis:** We evaluate the computational efficiency of our method by computing its average memory usage and inference latency across eight tasks and comparing these values against the corresponding baselines' averages.

Compared to the baseline DP3, our approach introduces minimal memory overhead while achieving higher success rates, demonstrating a favorable efficiency-performance trade-off for bimanual manipulation (Table 2).

**Table 2:** Model complexity comparison.

Model	Number of parameters(avg)	Initial memory (GB) (avg)	Peak Memory (GB) (avg)	Memory Increment (GB) (avg)	Avg.inference time (s) (avg)
DP3	262.4325 M	2.274	2.356	0.082	1.3981
OURS	265.6796 M	2.301	2.384	0.083	1.5214

**Figure 5:**
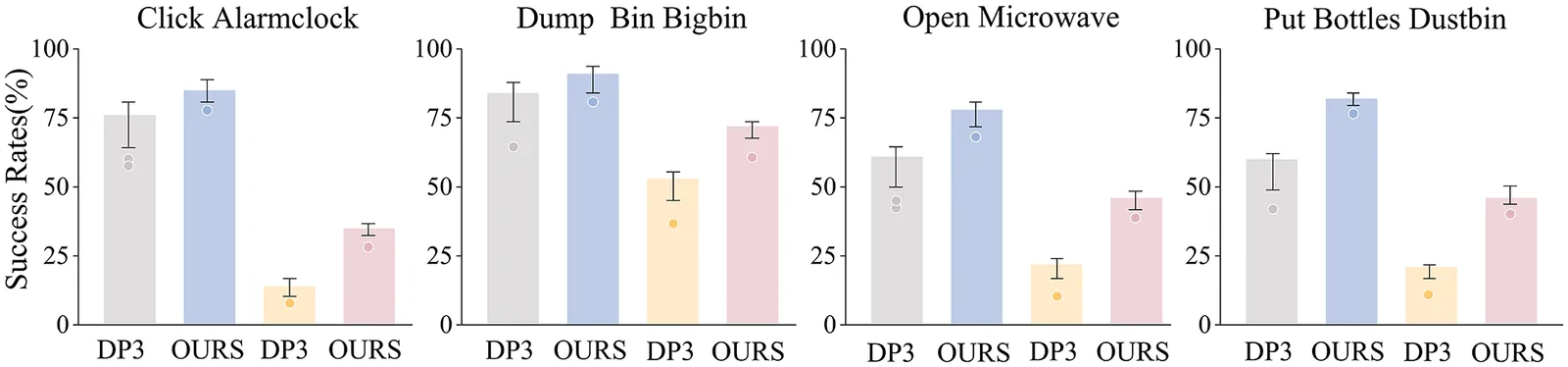
Task success rates of GSP-3D vs. DP3 on different tasks under both demo clean and demo randomized settings. For each task, the left and right bars indicate task success rates under the demo clean and demo randomized settings, respectively.

**Performance comparison under clean vs. domain-randomized conditions:** Figure 5 reports the task success rates under both the Demo Clean and Demo Randomized settings, where the Demo Randomized environment incorporates random lighting, cluttered backgrounds, and tabletop distractors, presenting a comparison between the baseline method and our method. The same evaluation environment is used across all conditions within each setting to guarantee fairness.

Across all tasks and both inference environment settings, our method consistently outperforms the baseline, achieving higher success rates, demonstrating superior robustness and effectiveness under both Demo Clean and Demo Randomized conditions.

**Task-level performance across different methods:** As summarized in Table 3, we further assess the efficacy of our proposed method by benchmarking it against several baseline approaches. All methods are evaluated under controlled experimental conditions, with consistent datasets, hardware configuration, and inference environment. For each configuration, multiple test runs are conducted, with each run consisting of 100 independent inference trials, and the reported success rates are averaged across all runs to ensure statistical reliability.

**Table 3:** Success rate (%) comparison of GSP-3D against all baseline methods across different methods and tasks.

Method	Click alarmclock	Handover block	Move CanPot	Place empty cup	Rotate QRcode	Scan object	Place fan	Press stapler
RDT	61%	45%	25%	56%	50%	4%	12%	41%
Pi0	63%	45%	58%	37%	68%	18%	20%	62%
ACT	32%	42%	22%	61%	1%	2%	1%	31%
DP	61%	10%	39%	37%	13%	9%	3%	6%
DP3	75%	70%	70%	65%	74%	31%	36%	69%
OURS	84%	78%	76%	74%	82%	40%	42%	76%

All baseline methods were implemented following their official default configurations provided by the RoboTwin 2.0 benchmark, including their standard hyperparameters, pre-processing pipelines, and evaluation protocols.

**Ablation study:** To validate the individual contribution of each key component in GSP-3D, we conduct an ablation study under the Demo Clean setting. All variants are trained and evaluated under identical conditions, using the same 50 expert demonstrations per task and the same evaluation protocol. For each variant, we run experiments with three different random seeds (42, 1,234, 2,024) and report the average success rate across seeds to account for training variability. The full GSP-3D consistently outperforms all ablation variants across every task, confirming the effectiveness of each proposed component (Table 4).

**Table 4:** Ablation study results under Demo Clean setting (average success rate across three random seeds, %).

Variant	Click alarmclock	Handover block	Move CanPot	Place empty cup	Rotate QRcode	Scan object	Place fan	Press stapler
GSP-3D (full)	84%	78%	76%	74%	82%	40%	42%	76%
W/o 3DGS	76%	72%	71%	66%	75%	32%	37%	71%
W/o WM	79%	75%	74%	68%	78%	36%	39%	72%
W/o both	75%	70%	70%	65%	74%	31%	36%	69%

## 5 Conclusion

We presented GSP-3D for bimanual manipulation, which unifies 3D Gaussian Splatting with diffusion policy learning and a predictive world model. Unlike prior methods that rely on sparse point clouds, our approach uses explicit 3D Gaussian primitives generated in real time by a Generalizable Gaussian Regressor. These primitives provide richer geometric conditioning for the diffusion policy, capturing surface orientation and volumetric occupancy that point clouds lack. Additionally, a transformer-based world model introduces an auxiliary regularization loss that enforces temporal consistency by penalizing physically implausible future scene transitions. This encourages the policy to generate actions that lead to coherent and realistic state evolution over time. Evaluated on RoboTwin 2.0 across diverse bimanual tasks, GSP-3D achieves superior success rates under both clean and domain-randomized conditions, with minimal computational overhead compared to baseline method.

Limitations include occasional failure cases under occlusion or dynamic tasks, sensitivity to the world model loss weight, degraded robustness under extreme lighting or reflective surfaces, unvalidated sim-to-real transfer, quadratic scaling of the world model with Gaussian count, and unsuitability for deformable objects, extremely long horizons, or high-frequency control. Future work includes evaluating GSP-3D on real-world hardware, extending the framework to handle dynamic objects and deformable manipulation tasks, incorporating online adaptation of the world model during deployment to improve robustness in unstructured environments, and integrating more principled physical inductive biases such as collision detection or rigid-body dynamics priors to enhance physical realism in complex interaction scenarios.

## Data Availability

The raw data supporting the conclusions of this article will be made available by the authors, without undue reservation.
